# Comparison of Lumbosacral Alignment in Geriatric and Non-Geriatric patients suffering low back pain

**DOI:** 10.12669/pjms.342.13969

**Published:** 2018

**Authors:** Burhan Fatih Kocyigit, Ejder Berk

**Affiliations:** 1 Dr. Burhan Fatih Kocyigit, Department of Physical Medicine and Rehabilitation, Kahramanmaras Sutcu Imam University School of Medicine, Kahramanmaras, Turkey; 2 Dr. Ejder Berk, Department of Physical Medicine and Rehabilitation, Kahramanmaras Sutcu Imam University School of Medicine, Kahramanmaras, Turkey

**Keywords:** Elderly, Low Back Pain, Lumbosacral Alignment

## Abstract

**Objective:**

Lumbosacral alignment is a crucial factor for an appropriate spinal function. Changes in spinal alignment lead to diminished body biomechanics. Additionally, lumbosacral alignment may affect quality of life, sagittal balance and fall risk in elderly. In this study, we aimed to compare lumbosacral alignment in geriatric and non-geriatric patients suffering from low back pain.

**Methods:**

A total of 202 (120 male and 82 female) patients who visited to physical medicine and rehabilitation clinic with low back pain between January 2017 and August 2017 were enrolled in this study. Standing lateral lumbar radiographs were obtained from the electronic hospital database. Lumbar lordosis angle, sacral tilt, lumbosacral angle and lumbosacral disc angle were calculated on lateral standing lumbar radiographs.

**Results:**

The mean age of the non-geriatric group was 43.02 ± 13.20 years, the geriatric group was 71.61 ± 6.42 years. In geriatric patients, lumbar lordosis angle, sacral tilt and lumbosacral disc angle were significantly smaller (p = 0.042, p = 0.017 and p = 0.017). No significant differences were observed in lumbosacral angle between the groups (p = 0.508).

**Conclusion:**

Our study indicates the specific changes in lumbosacral alignment with aging. Identifying these changes in lumbosacral alignment in the geriatric population will enable to create proper rehabilitation strategies.

## INTRODUCTION

The spine, pelvis, hip joints and lower extremities are continuously contact with each other to transmit the body weight to the ground with minimum energy and deformity. Lumbosacral alignment is one of the crucial factor for appropriate spinal function.^1^ Previous studies have highlighted the importance of lumbosacral alignment for providing a proper posture in the healthy population.^2^,^3^ Biomechanical studies have shown that; sagittal plan lumbar spine morphology and anatomical curvatures affect the pressure on intervertebral discs.^4^ Multiple etiological factors lead to variations in lumbosacral alignment. Low back pain changes the curvature degrees of the lumbosacral region.^5^ Patients with low back pain change their posture to cope with pain.^6^ Extension - mobilization therapy has been found to be beneficial in the case of acute low back pain.^7^ On the contrary, chronic low back pain patients especially with spinal stenosis benefit from flexion posture.^8^ It has been shown that the lumbar lordotic angle decreases in patients with lumbar disc herniation. Patients with lumbar discopathy reduce lumbar lordosis to avoid posterior disc hyperpression. This condition reduces pain level.^9^

Aging is another important factor which changes lumbosacral alignment. The lumbar spine is constantly changing and reshaping throughout life. Takeda et al.^10^ observed patients for 10 years in their study. They determined 7.7 degree decrement in lumbar lordosis angle (LLA) in their cohort. With aging, protein synthesis decreases in several tissues. This condition causes weakness in the muscles and ligaments. The fluid content of the cells decreases whereas the fragility of the tissues increases. Additionally, intervertebral disc degeneration contributes spinal changes.^11^ All these variations lead postural abnormalities in elderly.

In our study we aimed to compare lumbosacral alignment between geriatric and non-geriatric patients suffering low back pain. Secondary aim was to determine the additional effect of aging on lumbosacral alignment in low back pain cases. Discovering specific spinal changes in geriatric patients with low back pain will contribute to create suitable rehabilitation strategies.

## METHODS

Patients who visited to our clinic with complaint of low back pain between January 2017 and August 2017 were included in this study. Standing lateral lumbar radiographs were obtained from the electronic hospital database. Total of 941 patients 18 years old or above were identified in the hospital database. Two groups were formed according to age: geriatric group (65 years or above), non-geriatric group (between 18 and 64 years). Patients who had spinal surgery, spinal infection, spinal trauma, spondyloarthritis, spinal tumour, scoliosis, excessive vertebral fracture were excluded. Additionally, patients whose radiographs were not appropriate were also excluded. Following the application of exclusion criteria, radiographs of 202 patients were included in this study.

Measurement of the lumbosacral angles was evaluated on digitalized lateral radiographs by Enlil PACS System software. This software enables placing straight lines and calculating lumbosacral angles. Lumbosacral angle calculations were determined as mentioned below:

***Lomber lordosis angle (LLA)*** was measured from the intersection of two lines drawn from the upper edge of the L1 vertebra and the lower edge of the L5 vertebra.

***Sacral tilt (ST)*** was measured from the intersection of the lines tangent to the posterior sight of S1vertebra and the vertical line.

***Lumbosacral angle (LSA)*** was measured from the intersection of the lines drawn from superior endplate of S1 vertebra and the horizontal line.

**Fig. 1:**
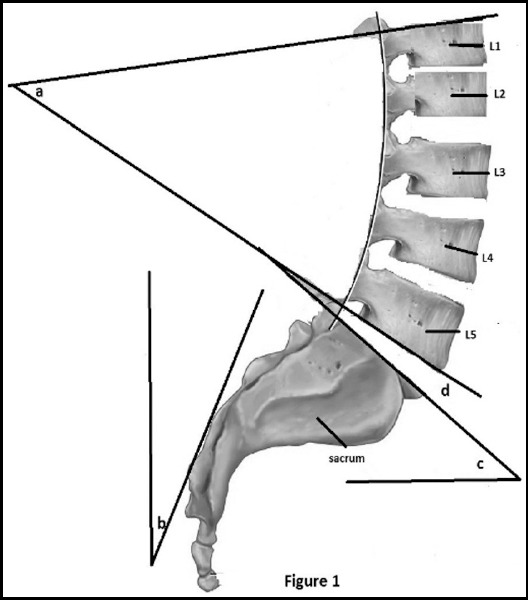
(a) Lomber Lordosis angle. (b) Sacral Tilt. (c) Lumbosacral angle. (d) Lumbosacral disc angle.

***Lumbosacral disc angle (LSDA)*** was measured from the intersection of two lines drawn from the inferior end plate of L5 vertebra and the superior end plate of S1 vertebra^12^,^13^ (Fig.1).

### Statistical analyses

Statistical analyses were evaluated with SPSS version 20.0 for Windows (SPSS Inc., Chicago, IL, USA). All results are expressed as mean value and standard deviation, number, percentage. Normality of the variables was analyzed with Shapiro-Wilk test. Chi-square test was performed to evaluate differences in categorical variables. Determination of differences in continuous variables between two groups was assessed using independent samples t-test. Pearson correlation analysis was used to determine the correlations between findings. The statistical significance value was accepted as 0.05. Correlation analyses were classified as weak, moderate, strong or very strong correlation according to coefficient (r) values being 0–0.25, 0.25–0.50, 0.50–0.75 or 0.75–1.00, respectively.

## RESULTS

A total of 202 patients were enrolled in our study. 122 patients (44 male, 78 female) were in non-geriatric group; 80 patients (38 male, 42 female) were in geriatric group. Gender differences were not statistically significant between groups (p = 0.106). The mean age of the non-geriatric group was 43.02 ±13.20 years, the geriatric group was 71.61 ± 6.42 years.

The mean values of the LLA, ST, LSA and LSDA were 40.25 ± 9.01, 40.62 ± 6.85, 38.39 ± 8.96 and 8.91 ± 2.51 in non-geriatric group. The mean values of the LLA, ST, LSA and LSDA were 37.73 ± 7.80, 38.32 ± 6.31, 37.50 ± 9.91 and 7.98 ± 2.89 in geriatric group. In the geriatric group, LLA, ST and LSDA were significantly smaller (p=0.042, p=0.017 and p=0.017). Despite that, there was no significant difference between the groups in terms of LSA (p=0.508) (Table-I).

**Table-I:** Lumbosacral Angles in Non-Geriatric and Geriatric group.

	Non-Geriatric Group (n = 122)	Geriatric Group (n = 80)	p-value
LLA (^0^)	40.25 ± 9.01	37.73 ± 7.80	0.042
ST (^0^)	40.62 ± 6.85	38.32 ± 6.31	0.017
LSA (^0^)	38.39 ± 8.96	37.50 ± 9.91	0.508
LSDA (^0^)	8.91 ± 2.51	7.98 ± 2.89	0.017

LLA: Lumbar Lordosis Angle, ST: Sacral Tilt, LSA: Lumbosacral Angle, LSDA: Lumbosacral Disc Angle.

When non-geriatric and geriatric group were pooled and analyzed according to gender; the mean values of the LLA, ST, LSA and LSDA were 36.95 ± 7.84, 37.88 ± 6.21, 36.19 ± 9.01 and 8.14 ± 2.79 in male patients. The mean values of the LLA, ST, LSA and LSDA were 40.82 ± 8.80, 40.95 ± 6.79, 39.30 ± 9.38 and 8.81 ± 2.62 in female patients. LLA, ST and LSA were found to be significantly higher in female patients (p=0.002, p=0.001 and p=0.02).No significant difference was observed between male and female patients in terms of LSDA (p=0.08).

### Non-geriatric group

The mean values of the LLA, ST, LSA and LSDA were 39.01± 8.36, 38.29 ± 6.16, 37.03 ± 8.45 and 8.21 ± 2.34 in male patients. The mean values of the LLA, ST, LSA and LSDA were 40.94 ± 9.33, 41.93 ± 6.91, 39.16 ± 9.19 and 9.30 ± 2.54 in female patients. ST and LSDA was significantly higher in female patients (p=0.004 and p = 0.022). No significant difference was found between male and female patients in terms of LLA and LSA (p = 0.258 and p = 0.208) (Table-II).

**Table-II:** Comparison of Lumbosacral Angles between Male and Female Patients.

	Non-Geriatric Group	P	Geriatric Group	P
**LLA(^0^)**				
Male	39.01± 8.36	0.258	34.56 ±6.51	<0.001
Female	40.94 ± 9.33		40.59 ± 7.83	
**ST(^0^)**				
Male	38.29 ± 6.16	0.004	37.42 ± 6.33	0.225
Female	41.93 ±6.91		39.14 ± 6.25	
**LSA(^0^)**				
Male	37.03 ± 8.45	0.208	35.23± 9.65	0.051
Female	39.16 ±9.19		39.55 ± 9.82	
**LSDA(^0^)**				
Male	8.21 ± 2.34	0.022	8.05±3.26	0.842
Female	9.30 ±2.54		7.92± 2.54	

LLA: Lumbar Lordosis Angle, ST: Sacral Tilt, LSA: Lumbosacral Angle, LSDA: lumbosacral disc angle.

### Geriatric group

The mean values of the LLA, ST, LSA and LSDA were 34.56 ± 6.51, 37.42 ± 6.33, 35.23 ± 9.65 and 8.05 ± 3.26 in male patients. The mean values of the LLA, ST, LSA and LSDA were 40.59 ± 7.83, 39.14 ± 6.25, 39.55 ± 9.82 and 7.92 ± 2.54 in female patients. LLA was significantly higher in female patients (p<0.001). However, there was no significant difference between male and female patients in ST, LSA and LSDA (p = 0.225, p = 0.051 and p = 0.842) (Table-II).

When the correlation of lumbosacral angles with each other were evaluated without dividing patients into two groups; LLA demonstrated a positive moderate correlation with ST (r=0.322; p<0.001). LSA showed a positive moderate correlation with LLA, ST and LSDA (r=0.322; p<0.001, r=0.489; p<0.001, r=0.267; p<0.001).

When the correlation of lumbosacral angles in the non-geriatric group was analyzed; LLA showed a positive moderate correlation with ST (r=0.386; p<0.001). LSA was positively and strongly correlated with ST and LLA (r=0.532; p<0.001, r=0.634; p<0.001); positively and weakly correlated with LSDA (r= 0.181; p<0.001).

When the correlation analyzes were evaluated in the geriatric group; LLA demonstrated a positive strong correlation with SIA (r=0.560; p<0.001). LSDA was positively and moderately correlated with ST and LSA (r=0.428; p<0.001, r=0.362; p=0.001).

## DISCUSSION

In our study, we analyzed lumbosacral alignment using lateral sagittal lumbosacral angles measurement. We compared measurements between two different groups; non-geriatrics and geriatrics. Additionally, differences were evaluated between male and female patients. All patients in our study had low back pain.

LLA, ST and LSDA were significantly smaller in geriatric group (p=0.042, p=0.017 and p=0.017). Furthermore, LSA was smaller in geriatric group but difference was not significant (p=0.508). Our results are consistent with previous studies. Takeda et al.^10^ observed 53 elderly volunteers for ten years in their study. During this period, 7.7 degree decrease was reported in the LLA which was statistically significant (p< 0.001). Imagama et al.^14^ included 100 male elderly subjects whose average age was 70 years in their study. Researchers evaluated LLA and sacral inclination angle using lateral standing radiographs and LLA was found to be significantly and negatively correlated with age. Merril et al.^15^ reported significant decrease in LLA and sacral slope (which is also named LSA) with aging. Asai et al.^16^ found a significant decrease in LLA with age. On the contrary, Yokoyama et al.^17^ did not find correlations between age and lumbosacral parameters. In another study, weak correlation was detected between age and sacral slope; no correlation was found between age and LLA.^18^ No significant correlations were observed between age and LLA, sacral slope in healthy adult volunteers.^19^ There may be various explanations of the different results in literature. Asymptomatic healthy subjects were evaluated in some researches. In healthy population, LLA must have increased to maintain spinal sagittal balance. This mechanism is deteriorated in patients with low back pain due to lumbar degeneration and disc collapse. This condition may explain the differences between asymptomatic population and patients with low back pain. Ethnicity may have played a role in these different results. Ethnicity was detected to have an impact on lumbosacral angles.^15^,^18^ Differences in radiographic technique and measurement method may also lead to the different results.

Along with age, remarkable changes occur in the spinal column and curvatures. More evident changes were observed after the age of 60 years.^11^ Several factors play a role in this situation. Weakness of back muscle strength, degeneration of discs and ligaments contribute to this change.^14^ Weakness of active and passive stabilizers of the spine lead to postural abnormality in the elderly.^20^ Age related changes of vertebra such as wedging, loss of height and compression fractures also play a role in this process. Rajnics et al.^21^ demonstrated that lumbosacral angles decrease in patients with lomber disc herniation which is more common in geriatrics. Changes of lumbosacral alignment occur due to avoidance of the sciatic nerve irritation in these patients. Additionally, analgesic flexion posture as a result of disc herniation leads to postural changes and decrement in lumbosacral angles.

Lumbosacral alignment LLA in particular, affects quality of life, sagittal balance and fall risk in geriatric population.^22^,^23^ Imagama et al.^14^ reported that LLA and sacral inclination angle decreases in elderly as a result of degenerative changes which cause an increase in thoracic / lumbar angle ratio. They demonstrated a negative correlation between thoracic / lumbar angle ratio and sagittal balance. Young adults decrease the thoracic kyphosis in order to compensate the changes in LLA. This compensation is insufficient in elderly as a result of non-flexible spinal column. This condition contributes to sagittal imbalance.^22^ Miyazaki et al.^24^ reported that LLA contributed to prediction of balance conditions. Additionally, proper spinal alignment was shown as an important factor for reducing fall risk in geriatric population.^25^

All our data were analyzed according to gender. LLA, ST and LSA were found to be higher in female patients. ST and LSDA were significantly higher in female patients in non-geriatric group and LLA was significantly higher in female patients in geriatric group. Our results have showed that female patients had a hyperlordotic posture. Similar to our results, Benlidayi et al.^26^ reported a hyperlordotic posture in female patients. Merril et al.^15^ demonstrated that female patients had higher LLA and sacral slope. In another study, female subjects were found to have higher LLA than male subjects.^16^ This may be a compensatory mechanism to anterior obstetric load on the spine in females.^27^ Contrary to our results some studies have found no relationship between gender and lumbosacral angles.^28^,^29^ Jannsen et al.^30^ demonstrated that female spine is different from male spine. However, they did not find any difference in lumbar lordosis between the genders. Yang et al.^18^ did not find any differences in lumbosacral parameters between female and male asymptomatic individuals. Cho^31^ reported that no significant differences were detected in LLA and sacral slope between female and male volunteers. These conflicting results may be a result of different measurement methods and ethnicity. Measurement errors, different sample size and selection bias may lead to this difference. Different imaging protocols in these studies may also contribute the conflicting results.

The relations of all these measurements with each other have an important role for maintaining proper spinal alignment. Any change in one of these parameters leads to a change in the others to provide functional and proper spino-pelvic unit. Thus, we evaluated the correlation analyses of lumbosacral angles. Boulay et al.^28^ demonstrated that a low value of pelvic incidence leads to a decrement in LSA which causes the flattening of lumbar lordosis. LSA balances the LLA.^32^ Similar to literature; we found a moderate positive correlation between LLA and LSA. Additionally, there was a positive strong correlation between LLA and LSA in non-geriatric group.

### Strengths

Results of this study describe how lumbosacral angles change with age and differ between sexes. Our study provides a better understanding and valuable informations on lumbosacral alignment. It is important for the physicians to be aware of the differences in lumbosacral parameters between these unalterable demographic characteristics. This study might also set light to future researches on the interventions preventing from malalignment in geriatric population. We believe that our results might help improving the treatment strategies of patients with spinal deformity.

### Limitations

It has a retrospective design. As a result of retrospective design, we could not evaluate the body mass index and occupation conditions. Hip joint, knee joint and ankle joint positions may influence the lumbosacral parameters. We could not evaluate these joints positions in our study. We could not assess the pain level and quality of life. Therefore, we could not identify clinical impacts of lumbosacral parameters. We could not compare the lumbosacral parameters between patients with acute low back pain and patients with chronic low back pain. We did not evaluate healthy controls. Our results must be confirmed with multi-centered prospective, longitudinal studies. Future longitudinal studies should clarify the association between lumbosacral alignment and clinical symptoms.

## CONCLUSION

This study has described the changes in lumbosacral alignment with aging. Our results are important for showing that age may influence the lumbosacral angles including LLA, ST and LSDA. LLA, ST and LSDA were smaller in geriatric patients. Variations in lumbosacral alignment have also been demonstrated among male and female patients. LLA, ST and LSA were higher in female patients. We consider that variations on lumbosacral alignment with aging and among sexes should be taken into account before the surgical plans and rehabilitation programmes. Strategies aiming to provide a good sagittal lumbosacral alignment need to be investigated. Studies with larger sample size in which standardized angle regions and methods are used are needed for determination of the changes in lumbosacral angles with age.

### Author`s Contribution

**BFK** conceived, designed and did statistical analysis & editing of manuscript.

**BFK, EB** did data collection and manuscript writing.

**BFK** did review and final approval of manuscript.

All the listed authors have approved the revised manuscript and is accountable for the integrity of this study.
